# Product Inhibition in Native-State Proteolysis

**DOI:** 10.1371/journal.pone.0111416

**Published:** 2014-10-31

**Authors:** Joseph R. Kasper, Elizabeth C. Andrews, Chiwook Park

**Affiliations:** Department of Medicinal Chemistry and Molecular Pharmacology, Bindley Bioscience Center, Purdue University, West Lafayette, Indiana, United States of America; University of South Florida College of Medicine, United States of America

## Abstract

The proteolysis kinetics of intact proteins by nonspecific proteases provides valuable information on transient partial unfolding of proteins under native conditions. Native-state proteolysis is an approach to utilize the proteolysis kinetics to assess the energetics of partial unfolding in a quantitative manner. In native-state proteolysis, folded proteins are incubated with nonspecific proteases, and the rate of proteolysis is determined from the disappearance of the intact protein. We report here that proteolysis of intact proteins by nonspecific proteases, thermolysin and subtilisin deviates from first-order kinetics. First-order kinetics has been assumed for the analysis of native-state proteolysis. By analyzing the kinetics of proteolysis with varying concentrations of substrate proteins and also with cleavage products, we found that the deviation from first-order kinetics results from product inhibition. A kinetic model including competitive product inhibition agrees well with the proteolysis time course and allows us to determine the uninhibited rate constant for proteolysis as well as the apparent inhibition constant. Our finding suggests that the likelihood of product inhibition must be considered for quantitative assessment of proteolysis kinetics.

## Introduction

Native-state proteolysis is a useful experimental approach to investigate transient partial unfolding in proteins under native conditions [1]–[4]. This method exploits the common observation that proteolysis of folded proteins requires partial unfolding. For a protease to hydrolyze a peptide bond in a protein, the substrate peptide chain needs to associate with the active site of the protease in a conformation that is productive for catalysis. Unless the substrate peptide chain is unstructured, proteolysis requires unfolding to a cleavable form in which the cleavage site is accessible to the protease for proteolysis [5]–[8]. The kinetic scheme of proteolysis coupled with unfolding is analogous to that used in native-state hydrogen/deuterium exchange:
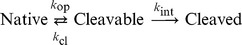
where *k*_op_ and *k*_cl_ are the rate constants for opening and closing of the cleavage site, and *k*_int_ is the intrinsic rate constant for proteolysis of the exposed cleavage site in the cleavable form [1]. When the proteolysis step is rate-limiting (*k*_int_<<*k*_cl_), pre-equilibrium can be approximated, and the observed rate constant for proteolysis (*k*_p_) is expressed as:
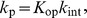
(1)where *K*_op_ is the equilibrium constant for the conformational change from the native form to the cleavable form. By determining *k*_p_ experimentally and approximating *k*_int_ from proteolysis kinetics of an unstructured peptide substrate by the same protease, one can determine *K*_op_. To avoid the bias from the sequence specificity of proteases in probing partial unfolding, we employ nonspecific proteases (e.g. thermolysin) in native-state proteolysis.

The rate constant of proteolysis (*k*_p_) is typically determined by monitoring the disappearance of an intact protein in a proteolysis reaction by SDS-PAGE [1], [9], [10]. Nonlinear curve-fitting of the plot of the band intensity versus time to a first-order rate equation gives a *k*_p_ value. The use of the first-order rate equation is based on the assumption that *k*_int_ is a pseudo-first-order rate constant that depends only on the protease concentration, not the substrate concentration [1]. This condition is satisfied when the enzyme is mostly free, i.e. the substrate concentration is much less than *K*_m_. Under this condition, *k*_int_ is expressed as *k*_cat_/*K*_m_[E_t_] in which *k*_cat_/*K*_m_ is the second-order rate constant for proteolysis of the cleavable form catalyzed by the protease and [E_t_] is the concentration of the protease [1]. However, we observed that *k*_p_ shows some dependence on the substrate concentration, indicating the first-order kinetics assumption is not fully satisfied in native-state proteolysis. We hypothesized that this deviation from the first-order kinetics is from inhibition of the nonspecific proteases by cleavage products. To test this hypothesis, we examined the proteolysis kinetics of two intact proteins *E. coli* dihydrofolate reductase (DHFR) and *E. coli* ribonuclease HI (RNase H) by two nonspecific proteases, thermolysin and subtilisin under various conditions. DHFR and RNase H have been separately verified as amenable to native-state proteolysis under our conditions [1], [11]. By assessing the effect of intact proteins and cleavage products on the apparent proteolysis kinetics, we confirmed that cleavage products are the inhibitory species. We also developed a kinetic model to explain the deviation from first-order kinetics by product inhibition.

## Results

### The apparent proteolysis rate constant is dependent on protein substrate concentration

When substrate concentration is low, the reaction catalyzed by an enzyme has pseudo-first-order kinetics, which can be described by a single exponential decay with an apparent first-order rate constant determined by *V*_max_/*K*_m_ or the product of *k*_cat_/*K*_m_ and the enzyme concentration. When we measure proteolysis kinetics of proteins by thermolysin, the typical concentration of protein substrates is 50 to 500 µg/ml (2.5 to 25 µM for a 20-kDa protein), which is much lower than the *K*_m_ values for proteolysis of peptide substrates by thermolysin (1–10 mM) [12], [13]. Moreover, in many cases proteolysis occurs through partially unfolded forms whose population is miniscule compared with that of native forms. Therefore, the protease is not likely to be saturated under our conditions for proteolysis.

**Figure 1:**
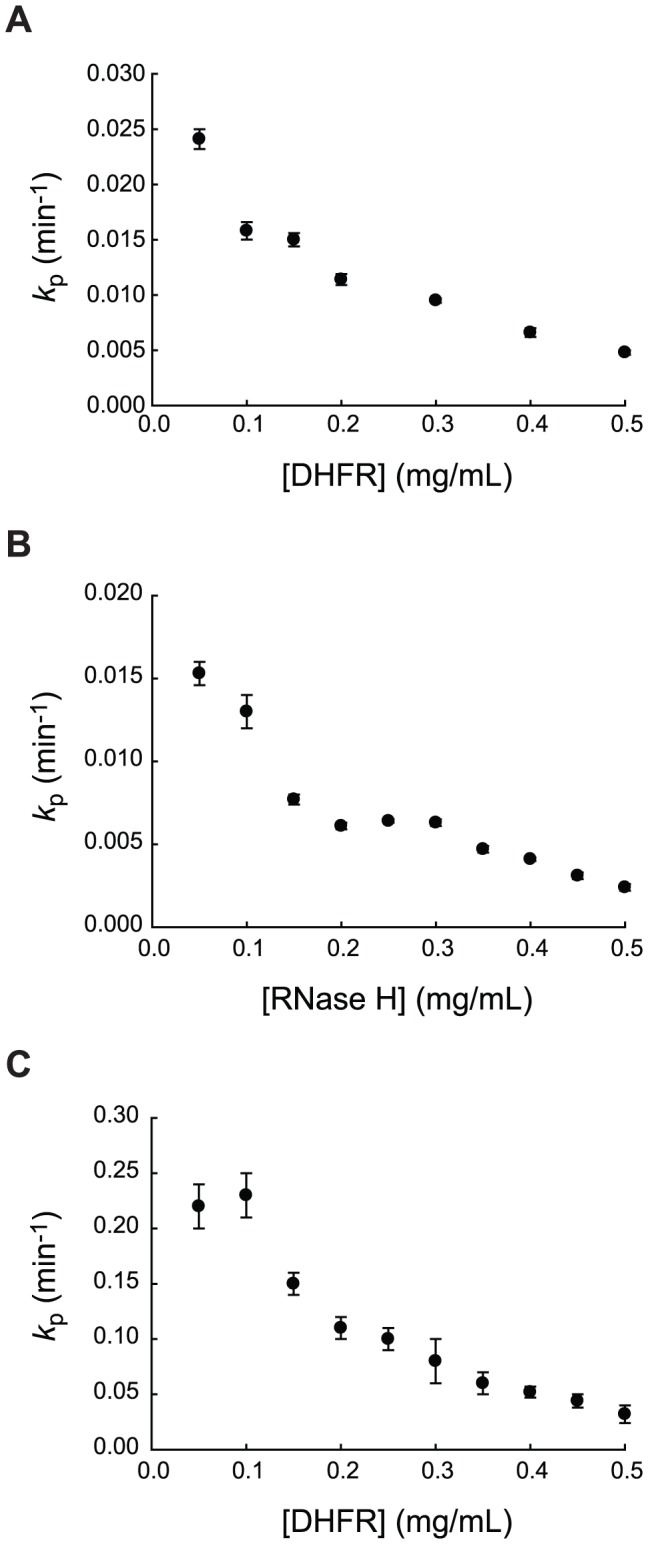
Dependence of the apparent proteolysis rate constants (*k*_p_) on concentration of substrate proteins. (A) Proteolysis of DHFR by 80 µg/ml thermolysin. (B) Proteolysis of RNase H by 200 µg/ml thermolysin. (C) Proteolysis of DHFR by 40 µg/ml subtilisin. Error bars show the standard errors from curve-fitting to a first-order rate equation.

To verify that our proteolysis condition satisfies pseudo-first-order kinetics, we determined the apparent first-order rate constants for proteolysis of DHFR by thermolysin at varying concentrations of DHFR (0.050–0.50 mg/mL) (Figure 1A). Interestingly, we observed that proteolysis of DHFR by thermolysin is slower with higher concentrations of DHFR. A ten-fold increase in DHFR concentration from 50 to 500 µg/ml resulted in a seven-fold decrease in the apparent rate constant. This dependence of the apparent kinetic constant on the substrate concentration suggests that the reaction does not satisfy the assumption of pseudo-first-order kinetics in which the apparent rate constant should depend only on the enzyme concentration. Fitting of the plot of remaining intact protein versus time to a first-order rate equation also shows non-random residuals, which indicates slight deviation from first-order kinetics (Figure S1).

To test if this deviation is specific to DHFR, we also explored the proteolysis of RNase H, which we have previously investigated using native-state proteolysis [1]. Again, a ten-fold increase in RNase H concentration (50 to 500 µg/ml) results in a six-fold decrease in the apparent rate constant (Figure 1B). This result shows that the deviation from the pseudo-first-order kinetics is not unique to DHFR. We then asked if other proteases also show this behavior. We monitored proteolysis of DHFR by subtilisin A at varying concentrations of DHFR. The substrate concentration we employed was much smaller than the known *K*_m_ values (0.50–58 mM) for subtilisin [14]. We found that the ten-fold increase in DHFR concentration resulted in a seven-fold decrease in the apparent rate constant (Figure 1C). These results clearly demonstrate that that proteolysis of intact proteins by nonspecific proteases does not fully satisfy pseudo-first-order kinetics under the employed experimental conditions. This deviation does not seem to be specific to the protease or the substrate.

### Cleavage products inhibit proteolysis

The apparent inhibition of the proteolysis reaction at higher substrate concentration may result from nonproductive binding with the substrate (substrate inhibition) or from binding with the cleavage products that accumulate during the reaction (product inhibition). Substrate inhibition results from formation of an enzyme-substrate complex that cannot react. Product inhibition, however, results from a stable enzyme-product complex that sequesters free enzyme as product develops. This enzyme-product complex may or may not be identical to the species formed following catalysis.

We first investigated the possibility of substrate inhibition for both proteases. We monitored proteolysis of a short fluorogenic peptide 2-aminobenzoyl-Ala-Gly-Leu-Ala-4-nitrobenzylamide (ABZ-Ala-Gly-Leu-Ala-NBA) in the presence and absence of 0.50 mg/mL DHFR. Both thermolysin and subtilisin digest the peptide substrate effectively. We monitored the increase in fluorescence upon cleavage to determine apparent rate constants for the proteolysis reaction. For both proteases, the effect of intact DHFR on the apparent rate constant is insignificant (Table 1). It is important to note here that the proteolysis of DHFR during this assay is negligible compared to the fluorogenic peptide. A folded protein is cleaved much more slowly than a peptide because it must unfold to be cleaved [1]. After 6-min incubation with 0.50 µg/mL thermolysin, less than 1% of intact peptide remains; however, even with 80 µg/ml thermolysin (Figure 1A), 97% of intact DHFR remains after six minutes.

**Table 1:** Effect of intact DHFR on the rate of proteolysis of a peptide substrate by thermolysin and subtilisin.

	Proteolysis by thermolysin	Proteolysis by subtilisin
	*k*_p_ (×10^−3^ s^−1^)	*k*_p_ (×10^−3^ s^−1^)
No DHFR	17 (±2)	5.4 (±0.1)
0.5 mg/ml DHFR	16 (±1)	5.1 (±0.1)

The rate of proteolysis of ABZ-Ala-Gly-Leu-Ala-NBA by thermolysin or subtilisin was determined in the presence and absence of intact DHFR. The rate constants (*k*_p_) were determined by fitting the change of the fluorescence intensity to a first-order rate equation. Values reported are the average of three replicates. Standard deviations are shown in parenthesis.

**Figure 2:**
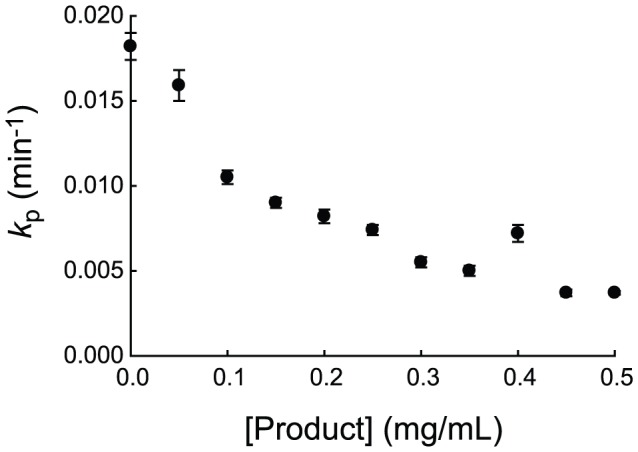
Effect of cleavage products on apparent proteolysis rate constants of DHFR by thrmolysin. Product was generated by incubating varying concentrations of DHFR with thermolysin until no intact DHFR was detectable by SDS-PAGE. Intact DHFR was then added to the resulting reaction to the final concentration of 0.050 mg/ml.

Because intact protein does not inhibit thermolysin, we investigated the effect of the cleavage products on the proteolysis kinetics. To generate products, we incubated various concentrations of DHFR with 80 µg/mL thermolysin for at least 18 hours, until intact protein was no longer detectable by SDS-PAGE. Subsequently, intact DHFR was added to the reaction at a final concentration of 50 µg/ml, and its digestion was monitored by SDS-PAGE. With a control experiment, we also confirmed that thermolysin does not lose activity significantly under this experimental condition (Data not shown). We found that the apparent first order rate constant for proteolysis of 50 µg/ml DHFR in the presence of 500 µg/ml cleavage products is five times smaller than for proteolysis of 50 µg/ml DHFR without added cleavage products (Figure 2). These experiments reveal that the products resulting from proteolysis of an intact protein are responsible for inhibition of thermolysin.

**Figure 3:**
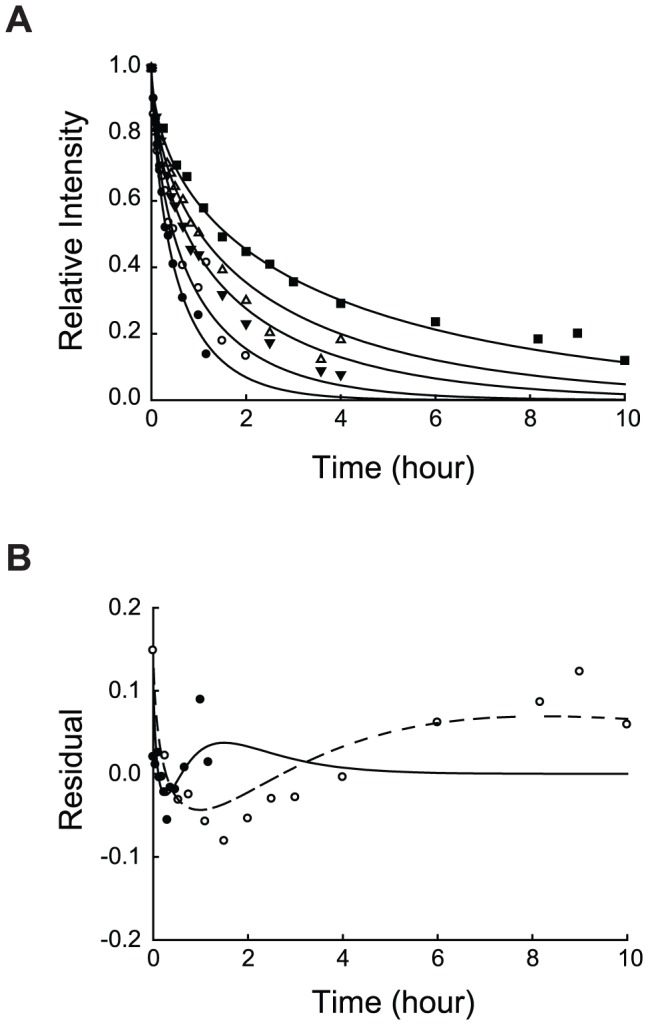
Determination of *k*_0_ and *K*_i_ by global fitting. (A) Fractions of intact protein remaining after proteolysis of 50 (▪), 100 (△), 200 (▾), 300 (○), and 500 (•) µg/ml DHFR by 80 µg/ml thermolysin are shown with the global fitting by Eq. 2. (B) Residuals are shown for fitting proteolysis of 50 µg/ml (•) or 500 µg/ml (○) DHFR by a first-order rate equation. Residuals are shown for fitting the proteolysis time courses predicted by global fitting parameters (solid lines in A) for 50 µg/ml (―) or 500 µg/ml (---) DHFR to a first-order rate equation.

Because the reaction products are clearly the inhibitory agent, we fit time courses of DHFR proteolysis (Figure 3A) using a competitive inhibition model:
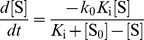
(2)


In this model, the reaction rate depends on the uninhibited rate constant, *k*_0_, the inhibition constant, *K*_i_, and the concentration of inhibitor ([S_0_]–[S]). [S_0_] is the initial concentration of the substrate protein, and [S] is the concentration of the remaining substrate at a given time. In our system, the molar concentration of inhibitor is unknown because we do not know the size of the cleavage products generated by proteolysis of DHFR. Also, it is likely that each cleavage product has a different ability to inhibit the reaction. As an approximation, we use µg/mL as the unit for substrate and inhibitor concentrations and a single *K*_i_ expressed in µg/mL. This *K*_i_ value is the concentration of the product in µg/mL at which the reaction rate is inhibited by 50% (IC_50_ in µg/mL) rather than an equilibrium constant.

We fit plots of the loss of substrate over time using a numerical solution to Eq 1 (Figure 3A). A global fit of time courses at five substrate concentrations gives an inhibition constant, *K*_i_, of 18±4 µg/ml and an uninhibited rate constant, *k*_0_, of 0.060±0.010 min^−1^. The value of *k*_0_ is significantly larger than the apparent rate constant even at 50 µg/ml DHFR (0.0242±0.0009 min^−1^). Apparently, proteolysis of 50 µg/ml DHFR generates a concentration of inhibitory products higher than *K*_i_ (18±4 µg/ml) at later time points, and the use of a first-order rate equation results in underestimation of the rate constant. We also fit the proteolysis time courses predicted by global fitting parameters to a first-order rate equation to test if the competitive inhibition model can predict the observed residuals. The predicted residuals agree well with the observed residuals (Figure 3B), confirming the validity of the model.

**Figure 4:**
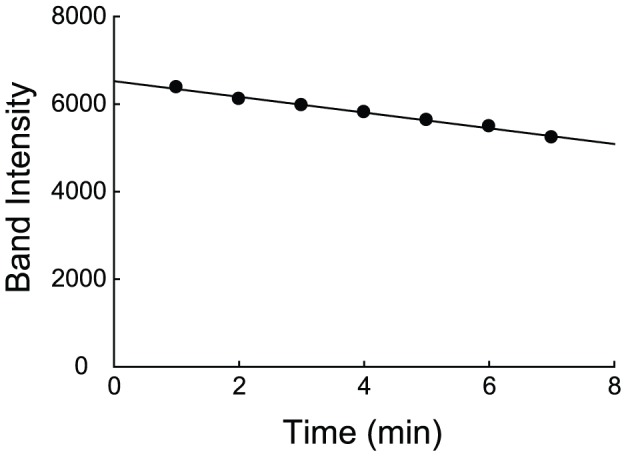
Initial velocity of proteolysis of DHFR by thermolysin. 100 µg/ml DHFR was incubated with 40 µg/ml thermolysin. The SDS-PAGE band intensity of intact DHFR is shown as a function of time. The solid line shows a linear regression.

We then sought to validate the uninhibited rate constant from the global fitting experimentally. Early in the reaction, product accumulation is minimal. Under this condition, the initial velocity of the reaction should approach that of the uninhibited reaction. To determine the initial velocity, we measured the loss of intact DHFR over time within the early linear region of the proteolysis reaction (Figure 4). About 80 percent of the intact protein remained at the latest time point. To slow the reaction, we reduced the thermolysin concentration to 40 µg/ml, which is half of the thermolysin concentration used to generate the data in the global fitting described above. The initial velocity was determined as 0.027±0.001 min^−1^, which is close to half that determined from the global fitting by the competitive inhibition model (0.06±0.01 min^−1^). As the rate constant is linearly proportional to protease concentration, the two rate constants are in very good agreement. The agreement of the initial velocity with *k*_0_ also confirms that the competitive product inhibition model is valid.

## Discussion

We have found that the deviation from first-order kinetics for proteolysis kinetics of intact proteins by thermolysin and subtilisin originates from inhibition by cleavage products that accumulate as proteolysis progresses. The inhibition by cleavage products can be modeled as competitive inhibition, with an uninhibited rate similar to the initial velocity of the proteolysis reaction. The identity of the inhibitory products is unknown. Most of the cleavage products generated by thermolysin and substilisin are likely short peptides with only a few amino acids because both proteases are nonspecific and can digest proteins at many locations. Though we use a single inhibition constant, *K*_i_, in our model, it is possible that multiple cleavage products inhibit the reaction with different levels of affinity.

Our results are consistent with the known properties of thermolysin. Thermolysin is a nonspecific protease but prefers substrates in which P1′ (the residue that contributes the amine group to the cleaved peptide bond) is contributed by a large hydrophobic residue [12], [15], [16]. A hydrophobic pocket that binds the P1′ side chain confers the protease with the preference for large hydrophobic groups at this site [17], [18]. Thermolysin tolerates proline at P1 (the residue that contributes the carbonyl to the cleaved peptide bond) but not at P2′ [19]. Further, a free amino terminus at P1 prevents cleavage [12], [16]. Likely because of these constraints to specificity, a number of dipeptides are known to inhibit thermolysin [20]. For example, the peptide Val-Trp binds with Val in the P1′ position and Trp in the P2′ position. In this arrangement, binding is achieved, but no cleavable bond is presented to the enzyme [18]. Such peptides in the product mixture would serve as inhibitors. The apparent product inhibition we observed in thermolysin may also result from inhibition by poor substrates. If two substrates are present that have equal affinity but different rates of turnover, the substrate with low turnover will act as an inhibitor for that with high turnover. The turnover number, *k*_cat_, for a series of thermolysin substrates spans 1000 fold [12]. Therefore, it is plausible that some peptides generated by proteolysis may act as inhibitors with respect to other peptides.

Several key properties of subtilisin provide evidence that is consistent with inhibition by short peptides. Subtilisin, like thermolysin is relatively nonspecific. Still, subtilisin preferentially cleaves peptides with Gly in P1′, a large hydrophobic residue in P2′ [19], and a large, non-β-branched hydrophobic residue in P1 [21]. Extending the substrate in the N-terminal direction up to at least four residues increases hydrolysis as well. The identity of residues at P2, P3 and P4 influence proteolysis, and the charged amino-terminus at these positions inhibits the enzyme [22]. Both bulky residues and residues carrying the carboxy-terminus in the P1′ position inhibit proteolysis [22]. These restrictions to subtilisin specificity also may lead to binding arrangements that do not result in catalytic turnover.

These results are consistent with observations of product inhibition in other proteases. Aminopeptidase P is inhibited by a number of cleavage products [23], [24]. The NS3 proteases of hepatitis C and dengue viruses, necessary for specific cleavage of the viral polyprotein, are both inhibited by their products [25], [26]. The protease, β-secretase, is inhibited by short peptides that appear to bind within the active site but have very slow turnover compared to the favored substrate [27].

Our findings have a clear implication to the use of native-state proteolysis in determination of the energetics of partial unfolding. The underestimation of *k*_p_ in Eq. 1 results in underestimation of *K*_op_ and Δ*G*_op_°. When *k*_p_ is determined to be 5-fold less than the true value, Δ*G*_op_° can be underestimated by ∼1 kcal/mol. The use of initial velocity might be a solution, but we have not fully tested the accuracy of this approach. Use of global fitting by the product inhibition model in Eq. 2 would be a valid method but requires multiple kinetic assays to generate the required data. Lowering the concentration of the substrate protein in the assay is the simplest way to minimize error from product inhibition. Another known source of error in the determination of *K*_op_ is the uncertainty in *k*_int_. When the initial cleavage site is known, one can determine *k*_int_ using a peptide substrate that contains a sequence identical to that of the cleavage site [1]. However, when the initial cleavage site for a protein by a given protease is unknown, *k*_int_ needs to be estimated from the proteolysis kinetics of a generic peptide substrate by the same protease [2]. Therefore, both the potential underestimation of *k*_p_ and the uncertainty in *k*_int_ are potential sources of the error in determination of Δ*G*_op_° in native-state proteolysis. Native-state proteolysis still offers a way to assess the accuracy of Δ*G*_op_°. At a relatively high urea concentration, but still lower than *C*_m_ (the midpoint of the transition in equilibrium unfolding), proteolysis may occur through globally unfolded state. Under this condition, Δ*G*_op_° is determined by the global stability of the protein (Δ*G*_unf_°). The similarity of Δ*G*_op_° to Δ*G*_unf_° confirms that the contribution of the errors in *k*_p_ and *k*_int_ to the determination of Δ*G*_op_° is not significant [1]–[3], [11]. Most of all, the potential errors in *k*_p_ and *k*_int_ are not a significant concern in the comparative use of Δ*G*_op_° in determination of the effect of urea (*m*-values) [1], [11] or the effect of mutations (φ_c_ values) [2], [11], which are the most valuable information that native-state proteolysis offers.

## Materials and Methods

### Preparation of proteins

Both DHFR and RNase H used in this study are cysteine-free variants. DHFR has C85A/C152S mutations [28], and cysteine residues in RNase H are all replaced by alanine [29]. These cysteine-free variants are well studied models for protein folding. DHFR and RNase H were expressed from transformed *E. coli* BL21(DE3) plysS cells grown to OD_600_ of 0.6 and induced with 500 µM isopropyl-β-D-thiogalactopyranoside (IPTG). DHFR was purified by DEAE Sepharose Fast Flow (GE Healthcare Life Sciences; Piscataway, NJ) anion exchange chromatography and Superdex 200 (GE Healthcare Life Sciences; Piscataway, NJ) size exclusion chromatography. RNase H was purified a described previously [28], [30]. Lyophilized thermolysin (Type X; Sigma-Aldrich; St. Louis, MO) was dissolved in 2.5 M NaCl, 10 mM CaCl_2_. Lyophilized subtilisin (Type VIII; Sigma-Aldrich; St. Louis, MO) was dissolved in water. Concentrations of all proteins were determined by absorbance at 280 nm using extinction coefficients determined according to their amino acid sequence [31].

### Proteolysis

Proteolysis of DHFR by thermolysin was initiated by adding thermolysin to varying concentrations of DHFR in buffer to achieve final conditions of 20 mM Tris-HCl (pH 8.0), 100 mM NaCl, 10 mM CaCl_2_, and 80 µg/ml thermolysin. At the desired time, 15-µl aliquots were quenched with 5 µl of 50 mM EDTA. An undigested control sample was prepared identically but without the addition of thermolysin. Proteolysis of RNase H was initiated by adding thermolysin to varying concentrations of RNase H in buffer to achieve final conditions of 20 mM sodium acetate (pH 5.5), 50 mM NaCl, 10 mM CaCl_2_, and 200 µg/ml thermolysin. At the desired time, 15 µl aliquots were quenched with 5 µl of 50 mM EDTA. Proteolysis of DHFR by subtilisin was initiated by adding subtilisin to varying concentrations of DHFR in buffer to achieve final conditions of 20 mM Tris-HCl (pH 8.0), 100 mM NaCl_2_, and 40 µg/ml subtilisin. At the desired time, 15 µl aliquots were quenched with 5 µl of 50 mM phenylmethylsulfonyl fluoride in isopropanol.

Quenched reaction samples were separated by 15% SDS-polyacrylamide gels. Gels were stained with SYPRO Red Protein Gel Stain (Life Technologies; Carlsbad, CA), and fluorescent images were taken with a Typhoon scanner (GE Healthcare Life Sciences; Piscataway, NJ). Intact protein gel bands were quantified from images with ImageJ. Apparent rates of proteolysis (*k*_p_) were calculated by fitting the change in band intensity over time to a first-order rate equation in OriginPro 8.5.1 (OriginLab; Northampton, MA). *k*_0_ and *K*_i_ for proteolysis of DHFR by thermolysin were determined from a global fit of the change of band intensity over time at five DHFR concentrations (50–500 µg/mL) (normalized to the band intensity of DHFR at t = 0) to Eq. 2 using Scientist 3.0 (Micromath; Saint Louis, MO).

### Proteolysis of fluorogenic peptide

Proteolysis of the fluorogenic peptide substrate, ABZ-Ala-Gly-Leu-Ala-NBA [32] was monitored by the increase of emission at 420 nm with excitation at 323 nm using a FluoroMax-3 fluorometer (Horiba Scientific; Edison, NJ). Proteolysis by thermolysin was monitored in 20 mM Tris-HCl (pH 8.0) buffer containing 100 mM NaCl, 10 mM CaCl_2_, 1.5 µM ABZ-Ala-Gly-Leu-Ala-NBA, and 0.50 µg/ml thermolysin with or without 0.50 mg/ml DHFR. Proteolysis by subtilisin was monitored in 20 mM Tris-HCl (pH 8.0) containing 100 mM NaCl, 1.5 µM ABZ-Ala-Gly-Leu-Ala-NBA, and 0.10 µg/ml subtilisin with or without 0.50 mg/ml DHFR. Rate constants for proteolysis were determined by fitting the change in fluorescence intensity over time to a first-order rate equation in OriginPro 8.5.1 (OriginLab; Northampton, MA).

### Proteolysis of DHFR in the presence of cleavage products

We prepared cleavage product by incubating varying concentrations of DHFR (0.050–0.50 mg/mL) with 80 µg/mL thermolysin until no intact DHFR was detectable by SDS-PAGE (18 hours for 0.05–0.10 mg/ml DHFR and 42 hours for 0.20–0.50 mg/ml DHFR). Intact DHFR was then added to the reactions to the final concentration of 50 µg/ml. Final conditions included 80 µg/ml thermolysin, 20 mM Tris-HCl (pH 8.0), 100 mM NaCl, and 10 mM CaCl_2_. Thermolysin alone was incubated for 42 hours as a control to check the loss of thermolysin activity during incubation. Apparent rates of proteolysis (*k*_p_) were calculated from fitting the change in the band intensity of intact DHFR over time to a first-order rate equation in OriginPro 8.5.1 (OriginLab; Northampton, MA).

### Initial velocity of DHFR proteolysis by thermolysin

To determine the initial velocity of the proteolysis of DHFR by thermolysin, 100 µg/ml DHFR was incubated with 40 µg/ml thermolysin in 20 mM Tris–HCl (pH 8.0) buffer containing 100 mM NaCl and 10 mM CaCl_2_. Initial velocity was calculated from a linear fitting of the remaining intact protein for the first seven minutes of the reaction.

## Supporting Information

Figure S1 **Deviation from first-order kinetics.** (**A**) Proteolysis of 500 µg/ml DHFR by 80 µg/mL thermolysin was monitored by SDS-PAGE. Bands corresponding to DHFR and thermolysin were marked accordingly. The lane labeled C shows the undigested intact DHFR, which was used as the time point at t = 0. Refer to panel B for the time of each lane. (**B**) The change in band intensity on the gel shown in (A) was fit to a first-order rate equation. Residuals from the curve-fitting are shown below.
(PDF)
