# Supplementary figures and images for: Product Inhibition in Native-State Proteolysis
Source: PLoS One. 2014 Oct 31;9(10):e111416. doi: 10.1371/journal.pone.0111416 (PMC4216078; doi:10.1371/journal.pone.0111416)

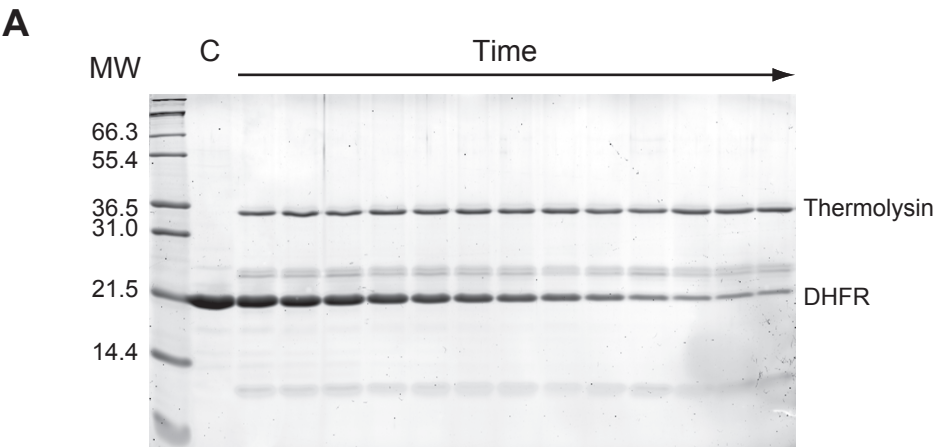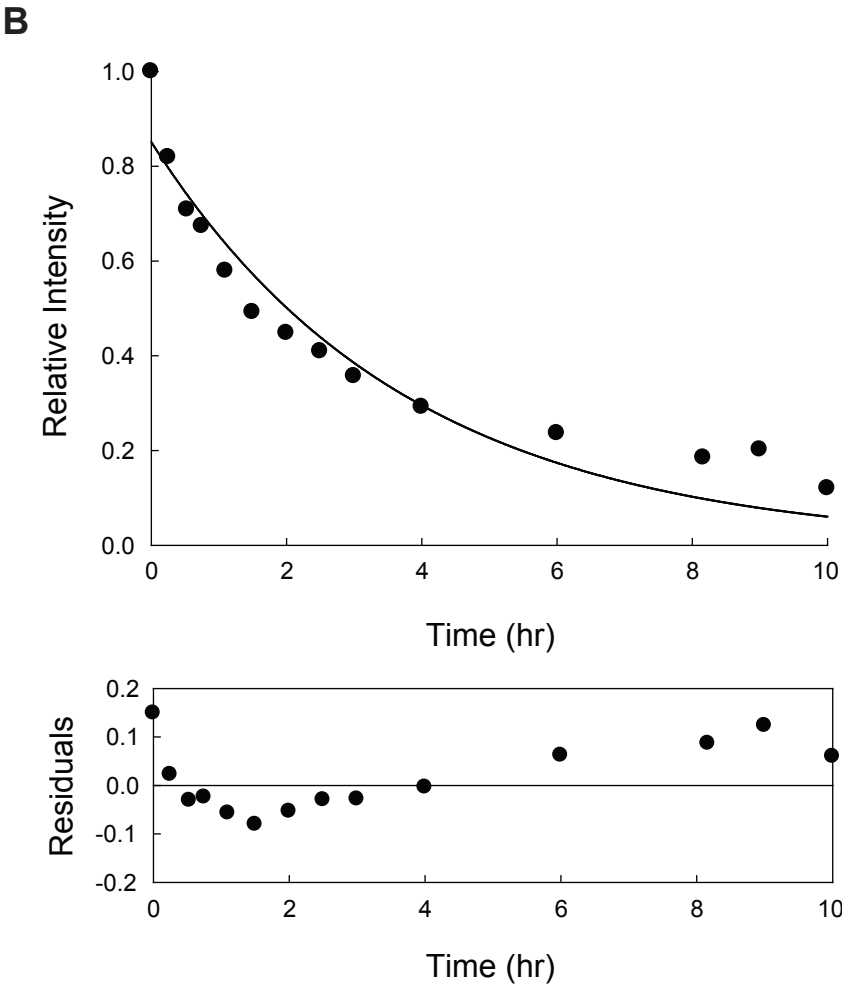

Supplement: Figure S1 — Deviation from first-order kinetics. (A) Proteolysis of 500 µg/ml DHFR by 80 µg/mL thermolysin was monitored by SDS-PAGE. Bands corresponding to DHFR and thermolysin were marked accordingly. The lane labeled C shows the undigested intact DHFR, which was used as the time point at t = 0. Refer to panel B for the time of each lane. (B) The change in band intensity on the gel shown in (A) was fit to a first-order rate equation. Residuals from the curve-fitting are shown below. (PDF) [file pone.0111416.s001.pdf]
